# Supplementary material for: Primary blast wave protection in combat helmet design: A historical comparison between present day and World War I
Source: PLoS One. 2020 Feb 13;15(2):e0228802. doi: 10.1371/journal.pone.0228802 (PMC7018002; doi:10.1371/journal.pone.0228802)
Supplement: Attachment — Submitted filename: Response to Reviewers.docx [file pone.0228802.s001.docx]

**Response to Reviewers**

Dear editor,
Dear reviewers,

In this letter, we address the reviewer comments for our submission of the manuscript “Primary blast wave protection in combat helmet design: a historical comparison between present day and World War I”

Kind regards,

Joost Op ‘t Eynde, Allen W. Yu, Christopher P. Eckersley, Cameron R. Bass
________________________________________________________________________

**The introduction was very interesting from a historic perspective; however, I think it could be condensed by about 30 to 50% while still retaining the essential historic information.**

Condensed historical section of the introduction. Overall introduction length reduced from 1162 words to 874 words (including new additions).

**You should include more clinical justification for spending this much effort analyzing primary blast (e.g. expand on your references 14 and 15). As you indicate, most such primary blast injuries result in so-called "mild" TBI as there is often no overt injury (e.g. subdural or penetrating brain injury). Some very interesting work from USUHS by MacDonald and Brody, et al. has demonstrated changes on MRI in such patients (PMID: 21631321). There is growing recognition that "mild TBI" results in significant morbidity over time. This should be more fully enumerated to justify this work and future efforts to improve helmet design such that it prevents both penetration (secondary) as well as primary blast injuries.**

Included
“In the past decade, there has been an increase in the awareness of long-term debilitating effects of primary blast mTBI, such as axonal injuries [16, 17]. Blast exposures causing minimal acute injuries might cause functional brain changes over time or with repeated exposures. Since most blast TBI is classified as ‘mild’, there is an increasing demand for combat helmets to protect against these exposures.”

**Did the helmets lose protective capacity with repeated blast exposure?**

There was no trend of decreasing protection with repeated exposure. At the highest exposure level, two of the helmets deformed as addressed in the results section. Added “For all other exposures, helmets experienced minimal deformation and no evidence of degradation by blast exposure was observed.”

**The underwash argument sounds very similar to the explanation some unformed individuals give for why seatbelts should not be worn--i.e. if I wear a seatbelt and am involved in a motor vehicle crash, I could be trapped in the wreckage. This has been shown to be patently false. Were you able to assess the validity of this underwash argument? Or did your methods preclude such an assessment. If it is the former, please enumerate. If the latter, consider deleting mention of "underwash."**

The focus on overhead blast in this study does not allow us to make a direct assessment on the validity of potential blast ‘underwash’. Section altered to “In other orientations, blast wave measurements are complicated by the difference between reflective (measured with pressure gauges oriented parallel to the direction of the blast) and incident (measured with pressure gauges oriented perpendicular to the direction of the blast) pressures, leading to conflicted reports of helmets possibly increasing the risk of primary blast injury [31-34, 41-43]. This risk has to be carefully evaluated, because reflected pressure measurements can be two to eight times greater than incident pressure measurements for the same blast scenario [37].”

**I disagree with the first sentence in your conclusions based on your introduction. It seems that primary blast protection was at least part of the motivation for designing helmets, at least in World War I. This may be less true today. Please clarify.**

Primary blast refers to damage caused by the shock wave resulting from the blast. Secondary blast describes injuries caused by projectiles energized by the blast such as shell fragments. Historical sources such as Dean 1920 provide shrapnel as the main threat addressed by the steel helmets, with some limited ballistic protection. Primary blast effects were poorly understood during World War I, and thus primary blast protection was not a design element.
Added clarification for the distinction between primary and secondary blast in introduction.

**Avoid the use of contractions (e.g. lines 433 and 513).**

Corrected.

**Sentence 425 does not make sense. "Helmets provided...."**

Corrected sentence order.

**Consider deleting the preamble "The authors want to emphasize...." in sentence 428.**

Removed.
